# Bridging the Gap in Understanding Behavioral Problems in Dementia: Insights from the Rythm Programme in India

**DOI:** 10.1002/alz70857_104977

**Published:** 2025-12-25

**Authors:** M Vaseel, Nalakath A. Uvais

**Affiliations:** ^1^ Iqraa International Hospital and Research Centre, Calicut, Kerala, India

## Abstract

**Background:**

Behavioral and psychological symptoms of dementia (BPSD) are a significant component of the dementia syndrome and affect up to 90% of patients throughout their illness, leading to poor outcomes. In India, there is limited information regarding the incidence, nature, course, and treatment of BPSD, particularly in rural settings. This study aims to address this knowledge gap by exploring these factors within a community treatment service context.

**Method:**

This study is conducted through a record review of 45 clinically diagnosed dementia cases from the Rythm Community Psychiatry Clinics in Kerala, India. The analysis specifically focuses on demographic factors, clinical presentations, the course of the illness, and treatment approaches related to behavioral problems associated with dementia.

**Result:**

The dementia cases presented with a variety of symptoms. Cognitive complaints were predominant, with forgetfulness reported as the primary issue in 81% of cases. Non‐cognitive symptoms included poor sleep in 63% of cases and sleep rhythm reversal in 18%. Behavioral symptoms were also significant in 60% of patients. Activity disturbances, such as wandering or inappropriate activities, were observed in 27% of cases. Affective disturbances were reported in 15% of cases. Additionally, psychotic symptoms such as delusions were present in 30% of cases, while visual hallucinations were noted in 12% and auditory hallucinations in another 12%. Other behavioral problems, such as echolalia, echopraxia, and disinhibited behavior, were seen rarely.

The average doses of pharmacological agents used included quetiapine at 40.38 mg/day, donepezil at 6.8 mg/day, memantine at 5 mg/day, melatonin at 3 mg/day, mirtazapine at 7.5 mg/day, and olanzapine at 2.5 mg/day. The most commonly reported side effects included dizziness, sedation, a tendency to fall, decreased food intake, and vivid dreaming.

Regarding the course of illness, 68% of cases responded well to interventions aimed at alleviating distressing behavioral symptoms. Common complications observed during the treatment course included recurrent hyponatremia, recurrent falls, urinary infections, and diminished ability to intake food. On average, patients received care from Rythm clinics for about 3.5 months.

**Conclusion:**

The Rythm Programme's findings highlight the significant prevalence of behavioral and psychological symptoms among dementia patients in the community, as well as the effectiveness of psychiatric interventions.

